# Unravelling the Role Played by Non-covalent Interactions in the Action Mechanism of PCDDs within Cells

**DOI:** 10.1021/acs.jcim.5c02555

**Published:** 2026-01-29

**Authors:** Lorena Ruano, Álvaro Pérez-Barcia, Vito F. Palmisano, Juan J. Nogueira, Marcos Mandado, Nicolás Ramos-Berdullas

**Affiliations:** † Department of Chemistry, 16722 Universidad Autónoma de Madrid, 28049 Madrid, Spain; ‡ Department of Physical Chemistry, 16784 University of Vigo, Lagoas-Marcosende s n, ES-36310 Vigo, Galicia, Spain; § Theoretical Chemistry Group, Zernike Institute for Advanced Materials, University of Groningen, 9747 AG Groningen, The Netherlands; ∥ Institute for Advanced Research in Chemistry (IAdChem), 16722 Universidad Autónoma de Madrid, 28049 Madrid, Spain

## Abstract

The aryl hydrocarbon receptor (AhR) is a ligand-activated transcription factor that mediates biological signals and regulates diverse cellular functions. Of particular concern are the effects triggered by dioxins and dioxin-like compounds (DLCs), whose toxicological outcomes arise through both canonical and noncanonical pathways, leading to the designation of AhR as the “dioxin receptor”. However, conventional risk assessment approaches based on toxic equivalency factors (TEFs), which primarily reflect the capacity of these compounds to bind and activate AhR, do not fully account for critical aspects such as environmental concentration and bioavailability, potentially underestimating their true impact. In this work, we present a comparative analysis of polychlorinated dibenzo-*p*-dioxins (PCDDs) with varying degrees of chlorination, focusing on their interactions with the AhR at the ligand-binding domain and on their permeation abilities across a model lipid membrane. To this end, we combine classical molecular dynamics (CMD) simulations with a hybrid quantum mechanics/molecular mechanics energy decomposition analysis (QM/MM-EDA) framework. This integrated approach enables a molecular-level characterization of receptor binding affinities and membrane permeation efficiencies. Our findings provide novel insights into the mechanisms underlying the relative toxicity of DLCs and highlight the need for integrative assessment strategies that encompass both receptor–ligand interactions and physicochemical behavior in biological environments. It is noteworthy that the toxicity of these compounds, as quantified by the pEC_50_ index, correlates with the membrane permeation barrier rather than with AhR binding affinity, identifying permeation as the key mechanistic step in the toxicological process of these compounds.

## Graphical abstract

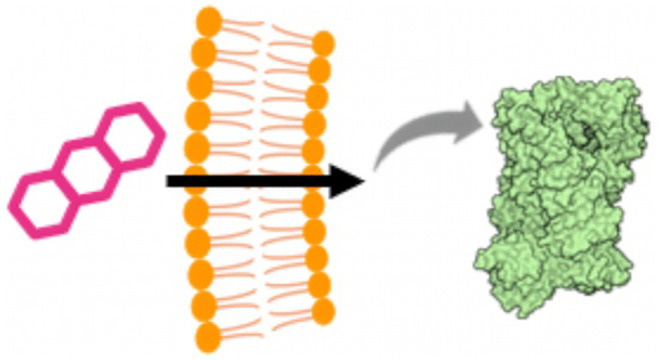

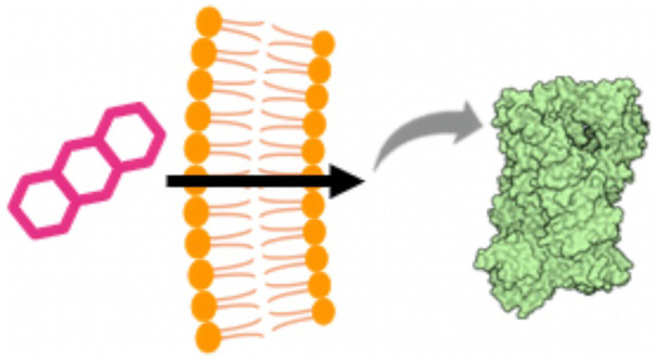

## Introduction

The aryl hydrocarbon receptor (AhR) is a cytosolic transcription factor characterized by ligand-dependent basic helix–loop–helix (bHLH) and Per–Arnt–Sim (PAS) domains.^1^ The AhR exhibits affinity for a broad spectrum of structurally diverse exogenous and endogenous ligands, including both agonists and antagonists.^2,3^ Upon activation, the AhR regulates the expression of numerous genes, including those involved in xenobiotic metabolism and others that influence key physiological processes^4,5^ and pathological conditions such as cancer.^6,7^ Specifically, the binding of dioxin-like compounds (DLCs), including polychlorinated dibenzo-*p*-dioxins (PCDDs), polychlorinated dibenzofurans (PCDFs), and polychlorinated biphenyls (PCBs),^8,9^ to the PAS-B domain of AhR initiates the dissociation of chaperone proteins and the formation of a heterodimer with the AhR nuclear translocator (ARNT). This complex translocates to the nucleus, where it binds to xenobiotic response elements (XREs) in deoxyribonucleic acid (DNA), promoting the transcription of metabolizing enzymes. This signaling cascade is widely recognized as the canonical AhR activation pathway.^10^

Despite this relatively detailed mechanistic understanding and nearly 50 years of research following the discovery of the high-affinity binding between the most toxic dioxin,^11^ 2,3,7,8-tetrachlorodibenzo-*p*-dioxin (TCDD), and AhR, which is often referred to as ”the dioxin receptor”, the scientific community continues to investigate the toxicological mechanisms and long-term health effects associated with chronic exposure to DLCs.^12–15^

Indeed, emerging evidence suggests that AhR signaling is not restricted to this canonical mechanism. Noncanonical pathways have been reported, varying by ligand structure, cellular context and environmental conditions.^16,17^ Moreover, several ligands are capable of eliciting toxic responses via AhR-independent mechanisms.^18^ These findings challenge the prevailing assumption that AhR activation alone fully accounts for the toxicity of DLCs and underscore the need for a broader understanding of the mode of action of the pollutants.^19^

In addition to receptor-mediated effects, the toxicological impact of dioxins is significantly influenced by their physicochemical properties as persistent organic pollutants (POPs).^9,20^ Routine toxicity assessment of DLCs is based on the toxic equivalency factor (TEF), which reflects their relative potency compared to TCDD and accounts only for AhR-mediated mechanisms. However, many DLCs with lower TEF values than TCDD are released into the environment in significantly larger quantities. Their high hydrophobicity, chemical stability and resistance to degradation lead to bioaccumulation, particularly in adipose tissues, facilitate biomagnification through the food chain and contribute to long-term toxic burdens in exposed organisms.^21,22^ Consequently, relying solely on TEF values may underestimate the actual risk posed by these compounds, as TEFs do not capture the effects of concentration and distribution.^8^ Therefore, a more comprehensive assessment of dioxin toxicity should integrate both receptor binding and physicochemical behaviors, including absorption, membrane transport and context-specific potency estimates beyond the TEF framework.^23^

The initial events involving both membrane transport and receptor engagement that underlie the mechanism of action of DLCs have rarely been investigated in detail at the molecular level. Early studies using molecular docking techniques provided valuable structural insights into the ligand-binding domain of AhR and identified key amino acid residues involved in TCDD binding.^24–26^ Advanced computational tools to capture receptor conformational flexibility, such as classical molecular dynamics (CMD) combined with enhanced sampling techniques have been used almost exclusively on TCDD in order to calculate binding energies and identify potential ligand-binding pathways to the human AhR.^27–29^ Similarly, studies of membrane absorption and diffusion processes have primarily used CMD simulations.^30–32^ In both receptor and membrane contexts, these classical methods previously employed lack the precision necessary to characterize the interactions with high accuracy, for which a quantum mechanical description would be beneficial.

To address these limitations, hybrid simulation approaches that integrate quantum mechanics (QM) for the interaction region with molecular mechanics (MM) for the surrounding environment have proven effective in accurately describing noncovalent interactions in complex biological systems. In particular, hybrid energy decomposition analysis (EDA) frameworks employing QM/MM calculations allow interaction energies to be partitioned into fundamental contributions: electrostatics, Pauli repulsion, induction and dispersion. Recent studies by the authors have successfully used this QM/MM-EDA approach to investigate the nature of intermolecular interactions in biologically relevant systems.^33,34^ Notably, one such study focused on the absorption and diffusion of two highly toxic compounds, TCDD and 2,3,7,8-tetrachlorodibenzofuran (TCDF), through lipid membranes.^35^

In the present study, a comparative analysis of PCDDs (see Figure 1) with different levels of chlorination was conducted and their interactions with AhR (see Figure 2A) and their permeation through a model lipid membrane were examined. To this end, a combination of CMD simulations and QM/MM-EDA was used to characterize these processes at the molecular level, complemented by the classical molecular mechanics generalized Born surface area (MM/GBSA) approach used for comparative analysis. The goal was to provide, for the first time, a deeper understanding, supported by accurate quantum chemical results, of the influence of PCDDs physicochemical properties on both receptor binding and membrane permeation, thereby offering new insights into the factors underlying their relative toxicity.

**Figure 1:**
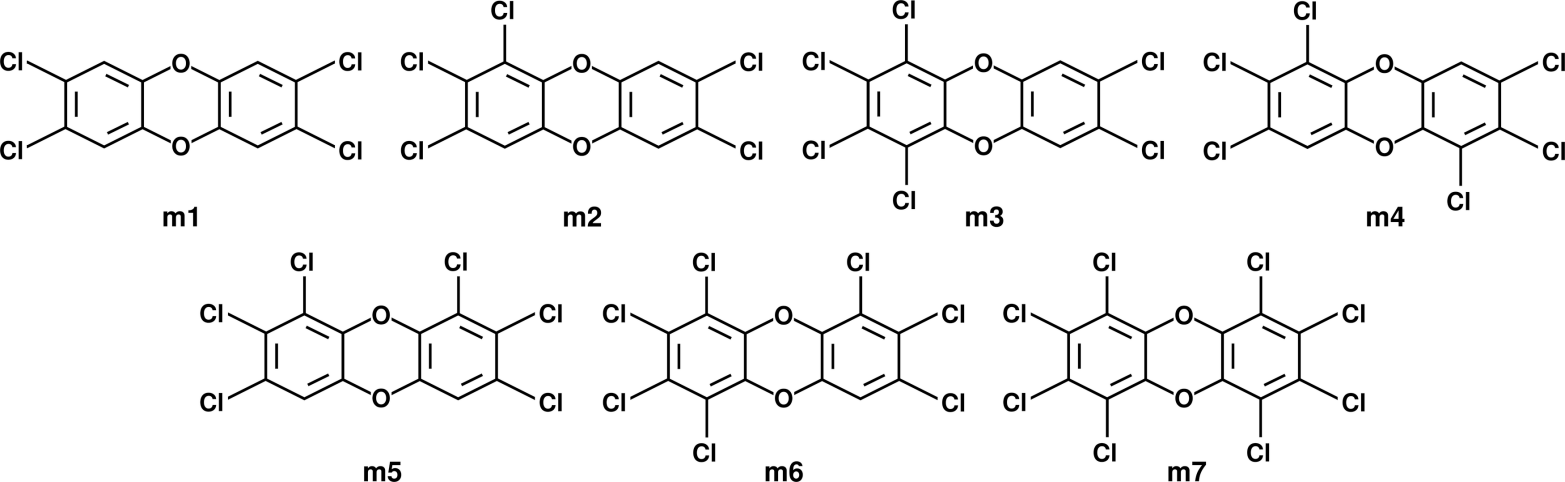
Polychlorinated dibenzo-*p*-dioxins (PCDDs) referred to as **m1** through **m7**.

**Figure 2:**
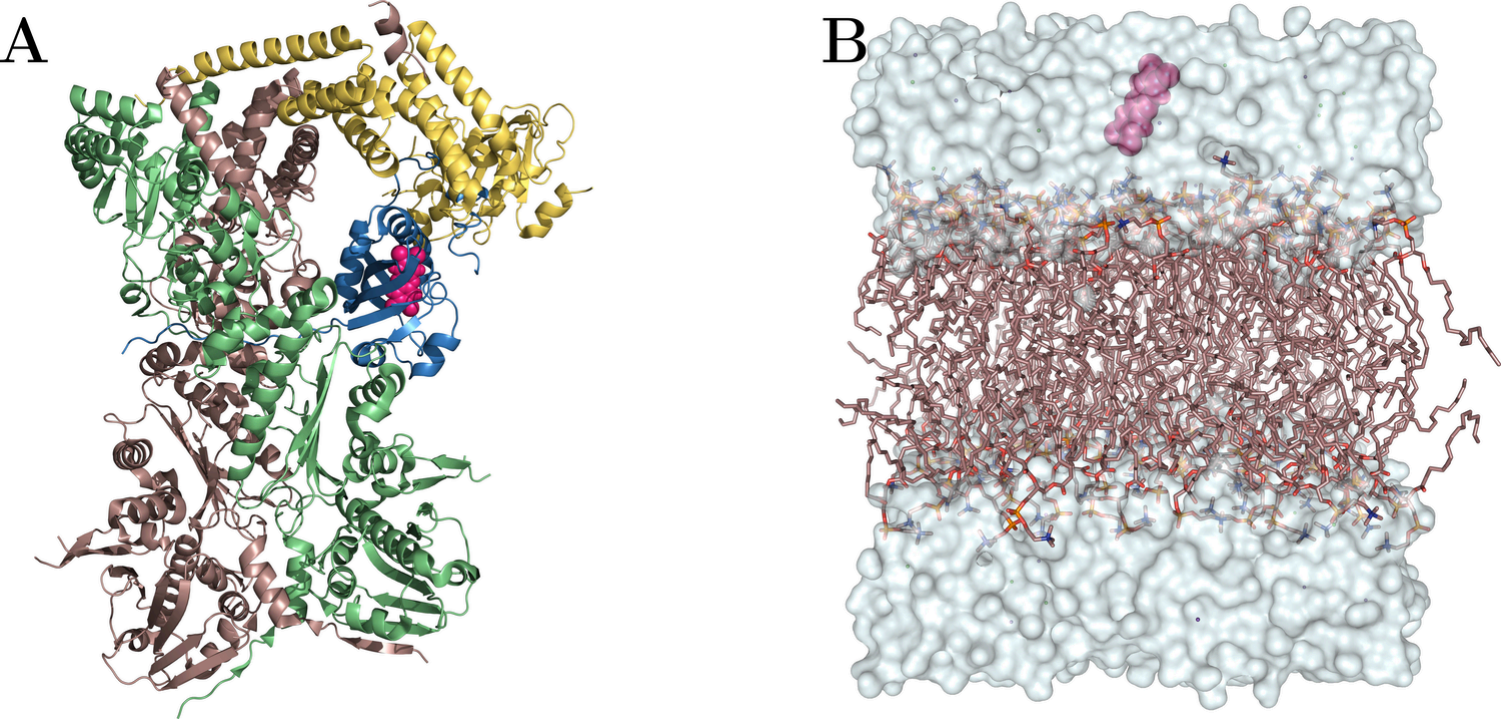
(A) AhR complex in blue with the chaperone Hsp90A in green, Hsp90B in salmon, the cochaperone XAP2 in yellow, and ligand in pink. PDB ID 7ZUB. (B) DOPC lipids in salmon, ligand at 32 Å in pink, and water in pale blue surface.

## Methodology and Computational Details

Prior to the CMD simulations, the structures of the PCDDs (see Figure 1), hereafter referred to as **m1** through **m7**, were optimized using density functional theory (DFT) with the B3LYP^36–39^ functional and the cc-pVDZ^40^ basis set. Additionally, Merz–Singh–Kollman charges were computed at the M06-2X^41^/cc-pVDZ^40^ level for subsequent use in the electrostatic component of the force field. CMD simulations were then performed with Amber20 ^42^ with the ligand integrated into the protein and the lipid membrane (see details below). After that, the interaction energy between the ligands and the protein and the membrane were characterized by two different methodologies: by the classical approach MM/GBSA and by a QM/MM-EDA scheme.

### Classical Molecular Dynamics Simulations

#### Lipid Membrane

A dioleoylphosphatidylcholine (DOPC) lipid bilayer composed of 64 DOPC molecules per layer was constructed using the CHARMM-GUI^43^ platform. A 22.5 Å thick water layer was added above and below the bilayer to solvate the system (see Figure 2B). To mimic physiological conditions, a 0.15 M KCl concentration was included. The membrane was described using the Lipid17 force field, an updated version of the earlier Lipid11 ^44^ and Lipid14 ^45^ force fields. The TIP3P model^46^ was used for water molecules, while K^+^ and Cl^–^ ions were described using appropriate Amber parameters.^47^ The system underwent a multistep relaxation protocol. Initially, 10000 steps of energy minimization were performed, transitioning from steepest descent to conjugate gradient halfway the simulation. This was followed by a two-step heating phase: first, a 5 ps NVT simulation increased the temperature to 100 K using a Langevin thermostat with a collision frequency of 1 ps^–1^; second, a 100 ps NPT simulation further heated the system to 303 K, applying a Berendsen barostat (2 ps pressure relaxation time) to maintain the pressure at 1 bar alongside the Langevin thermostat. During heating, a positional restraint of 10 kcal/(mol Å^2^) was applied to the lipid molecules. Subsequently, a 5 ns NPT equilibration run were performed to stabilize the periodic box dimensions. A 125 ns production simulation followed under the same NPT conditions as in the second heating step. Finally, a randomly selected snapshot from the production trajectory was extracted for use as the final system configuration.

After the equilibration of the lipid membrane, each PCDD molecule was positioned 32 Å above the center of mass of the relaxed DOPC membrane. Force field parameters for the PCDDs were derived from the general Amber force field^48^ (GAFF2), with Merz–Singh–Kollman charges computed at the M06-2X^41^/cc-pVDZ^40^ level of theory with Gaussian16 ^49^ software. The system then underwent a 20000-step energy minimization, transitioning from a steepest descent algorithm to conjugate gradient halfway the simulation. Subsequently, the system was heated to 303 K under NVT conditions using a Langevin thermostat with a collision frequency of 1 ps^–1^. Positional restraints of 10 and 5 kcal/(mol Å^2^) were applied to the lipid bilayer and the PCDD molecule, respectively, during this 1 ns heating phase. Following heating, four consecutive 5 ns equilibration runs were performed in the NPT ensemble to prepare the system for umbrella sampling simulations. These simulations employed a Berendsen barostat with a pressure relaxation time of 1 ps and the Langevin thermostat with a collision frequency of 1 ps^–1^ to maintain a pressure of 1 bar and a temperature of 303 K, respectively. During the equilibration process, positional restraints of 10.0, 5.0, 2.5, and 1.0 kcal/(mol Å^2^) on lipids and 5.0, 2.5, 1.0, and 1.0 kcal/(mol Å^2^) on PCDDs were applied, respectively.

After equilibration, each ligand was pulled along the axis perpendicular to the lipid bilayer (*z*-axis) toward the bilayer center to define the reaction coordinate, expressed as the distance between the centers of mass of the pollutant and the membrane along the *z*-axis. Subsequently, umbrella sampling simulations (32 ns per window) were performed to sample the configurational space of each ligand along this reaction coordinate. A pulling force constant of 1.1 kcal/molÅ^2^ and a pulling rate of 1 Å/ns were applied, while a restraint force constant of 2.5 kcal/molÅ^2^ maintained each PCDD within its respective sampling window (see the Supporting Information Figure S1). The resulting trajectory was divided into 32 windows along the reaction coordinate. For each window, a 30 ns CMD simulation was performed under the same conditions as the pulling step. The weighted histogram analysis method (WHAM) was employed to obtain the free-energy profiles, and the Monte Carlo bootstrap error analysis from WHAM was applied to calculate the statistical error.^50^ All simulations employed a 2 fs time step, with bonds involving hydrogen atoms constrained using the SHAKE^51^ algorithm. Long-range electrostatic interactions were treated with the particle-mesh Ewald^52^ (PME) method with a cutoff of 10 Å. At the bilayer center of each system, the total binding free energy (Δ*G*_tot_) was computed using the MM/GBSA method.^53,54^ The decomposition of Δ*G*_tot_ into van der Waals (vdW), electrostatic, polar solvation, and nonpolar solvation components was also evaluated (see eq 1, where *T*Δ*S* is neglected because is similar for analogue molecules). The first 5 ns of each trajectory were discarded for analysis (see Figure S2). A salt concentration of 0.15 M was used and solvation radii were assigned using the mbondi2 radii set (igb = 5), as developed by Onufriev et al.^55^

$$\Delta G_{\mathrm{tot}}=\underset{\Delta E_{\mathrm{int}}}{\underset{︸}{\mathrm{electrostatic}+\mathrm{vdW}}}+\underset{\Delta G_{\mathrm{solv}}}{\underset{︸}{\mathrm{polar}+\mathrm{nonpolar}}}-T\Delta S$$ 1

#### Aryl Hydrocarbon Receptor

The crystal structure of the protein with PDB ID 7ZUB was obtained from the Protein Data Bank. The AhR corresponds to chain D (residues 286–399) of this structure and was used for all subsequent simulations (see Figure 2A). Rigid molecular docking was performed with Autodock Vina software^56,57^ to determine the optimal binding pose of the ligands within the receptor’s binding site.^10,58^ A grid size of 16 × 16 × 16 Å^3^ with a grid spacing of 1 Å, the box centered at 160.5, 164.2, and 159.4, and an exhaustiveness of 8 were used. The pose with the most favorable free energy was then solvated in a truncated octahedral water box, ensuring a minimum distance of 14 Å between any protein atom and the box edge and Na^+^ and Cl^–^ ions were added using the *tleap* module of AmberTools20.^42^ The system components were described as follows: the protein with the ff19SB force field,^59^ the ligand with GAFF2,^48^ the water molecules using the TIP3P model,^46^ and ions with parameters developed by Joung and Cheatham.^47^

Then, in order to equilibrate the system structure and density, CMD simulations were carried out. Energy minimization was first performed using 5000 steps conducted by the steepest descent approach, followed by 5000 steps using the conjugate gradient method. The system was then gradually heated from 0 to 303.15 K over 40 ps in the NVT ensemble, using a Langevin thermostat with a collision frequency of 1 ps^–1^. Finally, a 500 ns production simulation was run in the NPT ensemble, applying a Berendsen barostat to maintain the pressure at 1 bar. The first 20 ns of the production are employed as equilibration during which the density of the systems and the protein structure get equilibrated (see below). A 2 fs time step was used throughout all stages. Electrostatic interactions were treated with the PME^52^ method, using a 12 Å cutoff. All bonds involving hydrogen atoms were constrained using the SHAKE algorithm.^51^

The total binding free energy of each of the ligands with the protein was calculated using the MM/GBSA approach. After an initial 20 ns period in which the protein structure stabilized, as assessed by root-mean-square deviation (RMSD) analysis, 200 evenly spaced snapshots were extracted from the remaining 480 ns of the production trajectory. These geometries were used to compute Δ*G*_tot_ and its decomposition into vdW, electrostatic, polar solvation, and nonpolar solvation components. A salt concentration of 0.15 M was used, and mbondi2 solvation radii were applied (igb = 5) following the model developed by Onufriev et al.^55^ Additionally, a pairwise ligand–residue energy decomposition analysis was performed to identify individual contributions from protein residues within 5 Å of each ligand’s center of mass. This analysis provided detailed insight into the energetic contributions of specific amino acids to ligand binding.

### Quantum Mechanics/Molecular Mechanics Energy Decomposition Analysis

In order to properly account for quantum effects, such as Pauli repulsion and dispersion forces, QM/MM calculations were performed with Gaussian16 ^49^ on a selected set of geometries, equally spaced along the CMD simulations. Specific details regarding the geometries selected for both membrane permeation and AhR binding are provided below. For the QM/MM calculations, an additive scheme was adopted, in which the Hamiltonian term describing the interaction between the QM and MM regions was represented through an electrostatic embedding scheme. We employed an electrostatic embedding rather than a polarizable one because previous studies have shown that, for a converged QM region size, the interaction energy and its individual components differ only marginally between the two approaches.^60^ Moreover, the slight reduction in QM region size that a polarizable embedding might enable does not offset the substantial increase in computational cost associated with computing the MM atomic dipoles required by the polarizable scheme. Subsequently, an EDA of the intermolecular interaction energies was carried out on these geometries, along with a statistical analysis of each energy component. The EDA scheme employed in this study follows the methodology developed by Mandado et al.,^61–63^ which is based on the fragmentation of the complex’s deformation density into Pauli and polarization contributions. Specifically, its extension to the QM/MM level, recently implemented in the EDA-NCI program^63,64^ was used. In this methodology, the different energy components include contributions arising from interactions between the QM and MM regions. In summary, the intermolecular interaction energy, *E*_int_, between the various PCCDs and either the membrane or the protein, is decomposed according to the eq 2

$$E_{\mathrm{int}}=E_{\mathrm{elec}}+E_{\mathrm{Pau}}+E_{\mathrm{ind}}+E_{\mathrm{disp}}$$ 2

where *E*_elec_, *E*_Pau_, *E*_ind_, and *E*_disp_ represent the electrostatic, Pauli, induction, and dispersion energy components, respectively. A detailed explanation of this EDA scheme, including mathematical derivations and applications at the QM/MM level, can be found elsewhere.^61–63^

#### Lipid Membrane

A series of equally spaced geometries were extracted from the window at the center of the membrane for subsequent quantum calculations. Single-point QM/MM computations, followed by the EDA calculations, were then performed on each selected geometry. These calculations employed the M06-2X-D3 ^41,65^ functional with the cc-pVDZ^40^ basis set for the QM region, while the interaction with the environment was described by an electrostatic embedding scheme.

For the EDA, the system was partitioned into two fragments: the PCDD molecule and the remainder of the system. Prior to choose the size of the QM region, a convergence analysis was conducted on a randomly selected geometry of the **m1** ligand system by increasing the number of QM residues until all energy components converge (see Figure 3A). Since the free energy minimum along the permeation pathway, formally called as potential of mean force (PMF), for each PCDD was located near the bilayer center, the chosen QM residues consisted of the hydrocarbon lipid chains closest to the ligand, introducing hydrogen as a link atom. Hydrogen link atoms were introduced when only the hydrophobic or hydrophilic parts of a lipid residue was included in the QM region. As shown in Figure 3A, convergence was achieved with 10 lipid residues included in the QM region.

**Figure 3:**
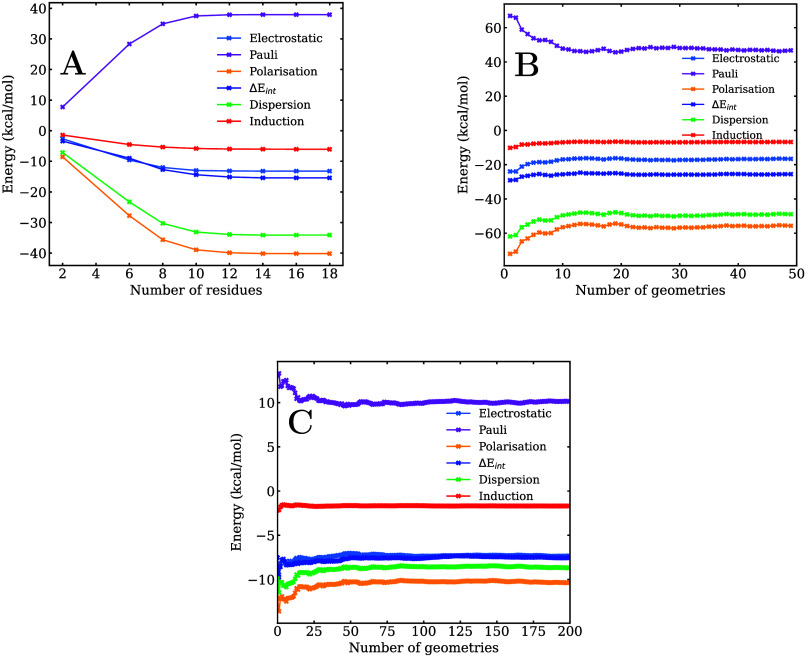
Convergence analysis of the **m1**/membrane total interaction energy and its different contributions with respect to the number of residues in the QM region (A) and the number of geometries along the trajectory with 10 lipid residues in the QM region (B). Convergence analysis of the **m1**/protein total interaction energy and its different contributions with respect to the number of geometries along the trajectory (C).

Additionally, an analysis was performed for **m1** to determine the minimum number of sampled geometries required for convergence of all energy terms. In these QM/MM calculations a QM region including 10 lipid molecules was employed. Figure 3B illustrates the behavior of the interaction energy and its components as a function of the number of sampled structures; the plot represents the mean value of each energy component. Convergence was observed for all energy terms when 34 geometries were used, and this number was therefore adopted for the final EDA. This converged number of residues and geometries was used for the rest of the systems.

#### Aryl Hydrocarbon Receptor

To reduce computational cost and complexity in the QM/MM investigation of PCDD affinity for the AhR, we adopted an inverse approach to that used for the lipid membrane. Specifically, we first assessed the number of geometries required to achieve convergence of the interaction energy components, using a minimal QM region. Thus, the QM region, treated at the M06-2X-D3 ^41,65^ /cc-pVDZ^40^ level with electrostatic embedding, included the smallest PCDD molecule of the series (**m1**) and the PHE295 residue, identified via MM/GBSA as contributing most significantly to the binding free energy. The MM region comprised chain D of the AhR, solvent, and ions. Mean interaction energy components were analyzed as a function of the number of geometries sampled (Figure 3C). Convergence was observed with 50 geometries, which were consequently adopted for subsequent EDA using an extended QM region. This extended region included the ligand and the 10 amino acid residues with the largest interaction energies from the MM/GBSA analysis, which should provide a large enough QM region to get converged results: HIS291, PHE295, TYR322, ILE325, CYS333, HIE337, ILE349, PHE351, LEU353, and VAL381. Notably, the top seven residues align with experimental observations from mutagenesis analyses.^58^ To address the covalent bonds cut when defining the QM and MM regions, we employed, as in the lipid membrane calculations, the link-atom approach, saturating the truncated QM bond with a hydrogen atom.

## Results and Discussion

### Membrane Translocation

The free energy profile along the reaction coordinate, defined as the axis perpendicular to the lipid bilayer, is presented in Figure 4 for the seven PCCDs studied. Before a careful analysis of the results, it is important to note that all the ligands investigated here are of considerable size. It has been shown that single runs of umbrella sampling simulations can provide inaccurate free-energy profiles, especially for large size systems.^66^ Accordingly, the following discussion is intended to be considered solely from a qualitative standpoint although histogram overlap and convergence analyses (see Figures S1 and S2) indicate an adequate behavior of the computed profiles. All molecules exhibit a pronounced decrease in free energy when transitioning from the aqueous phase outside the membrane to the bilayer’s central region, where the PCCDs are predominantly surrounded by the nonpolar tails of the DOPC molecules. This energy decrease ranges from −9 kcal/mol (**m1**) to −14 kcal/mol (**m6**) and two distinct behaviors can be identified inside the bilayer: while **m1**–**m5** show a flat region at the membrane center, **m6** and **m7** display a maximum that separates two minima located at around 7 and −7 Å. These energy minima for **m6** and **m7** are energetically located at −1.07 and −1.33 kcal/mol, respectively, relative to the energy at the membrane center.

**Figure 4:**
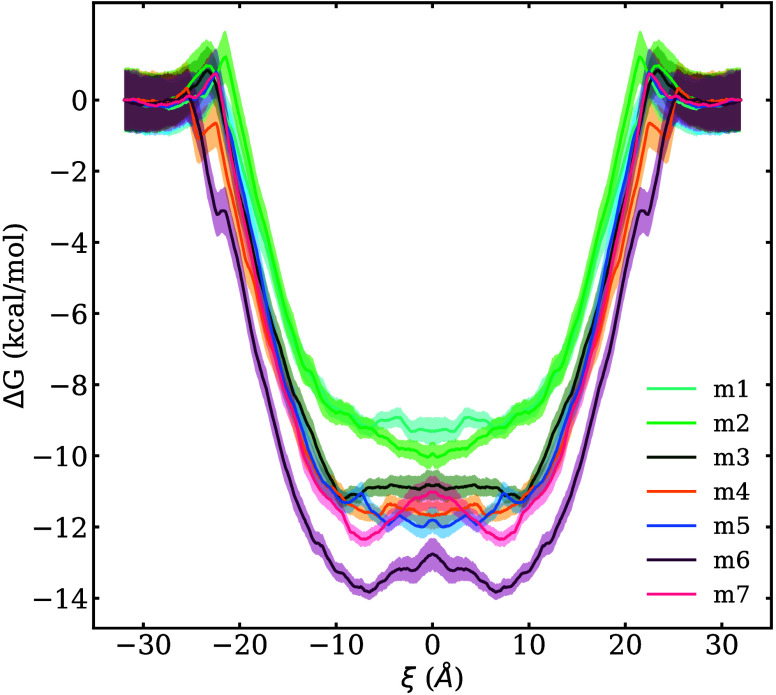
Potential of mean force of the membrane permeation process for all the ligands computed from 5 to 30 ns along the reaction coordinate from 32 to 0 Å. It is assumed that the profile is symmetric from 0 to −32 Å. Shaded areas represent the error.

Figure 4 shows a general trend of decreasing free energy at the minima with increasing chlorination, except for **m7**, which exhibits a higher free energy than **m6**. Such a deviation from the observed trend may originate from the limitations of umbrella sampling in systems with complex free energy landscapes and complex reaction coordinates that include different degrees of freedom, which are particularly relevant for large and flexible permeants, being the former the present case. This general trend is corroborated by the calculation of the binding free energy and its decomposition into electrostatic, vdW and solvation components at the center of the bilayer by using MM/GBSA analysis. The corresponding results are presented in Figure 5A and Table S1. As can be observed, both the interaction energy between the ligand and the membrane, Δ*E*_int_, and the total free energy, Δ*G*_total_ (including solvation effects), decrease (become more negative) with increasing chlorination without exceptions. Accordingly, **m1** exhibits the least negative free energy, followed by **m2**, **m5**, **m3**, **m4**, **m6** and finally **m7**. It is worth noting that **m3**, **m4** and **m5** share the same degree of chlorination and, thus, similar interaction and binding free energies.

**Figure 5:**
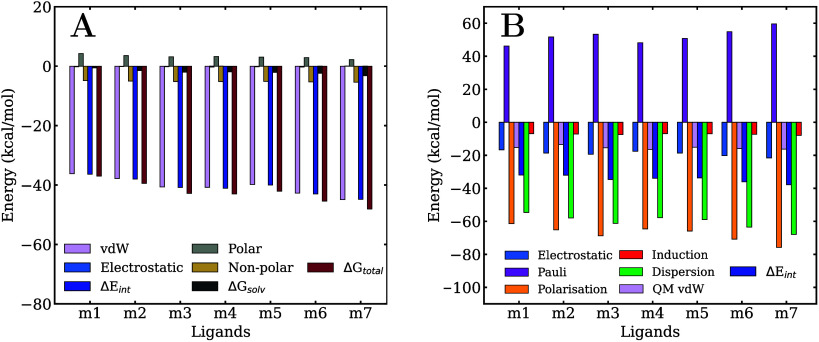
Decomposition of the ligand/membrane (A) MM/GBSA binding free energy and (B) QM/MM-EDA total interaction energy.

On the other hand, the separate analysis of the electrostatic and vdW components of the ligand/membrane interaction energies presented in Figure 5A and Table S1 reveals a negligible contribution from electrostatic forces. In all cases, their absolute values are below 0.4 kcal/mol and, in the case of **m7**, even slightly repulsive. Consequently, the vdW forces account for nearly 100% of the total interaction energy. This can be rationalized by considering that the minima of the free-energy profiles are located in the nonpolar region of the membrane and, thus, non-electrostatic interactions dominate the systems. However, QM calculations are needed to corroborate the conclusions extracted from the classical analysis.

The probability of PCDDs binding to their target protein AhR is proportional to their intracellular concentration, which is, in turn, dictated by the thermodynamics of membrane permeation. This permeation process can be conceptually divided into two sequential thermodynamic steps. In the first step, the compounds diffuse from the extracellular aqueous phase into the lipid bilayer, a process that is highly thermodynamically favorable across the PCDD congeners. The second step involves the translocation of the molecules accumulated in the membrane into the intracellular aqueous environment; this step is highly thermodynamically unfavorable, as the free energy well identified within the inner region of the membrane during the translocation process functions as an energetic barrier that limits the penetration of pollutants into the intracellular compartment. Hence, the greater the depth of this well, the lower the ability of the pollutants to exert their toxicological effects. On the other hand, the toxicity of PCDDs is commonly quantified using the pEC_50_ index, defined as the negative logarithm of the EC_50_ value, which is the extracellular concentration of the pollutant required to elicit 50% of the maximal toxic effect.^8,9^ Given that membrane permeation strongly influences the intracellular accumulation of PCDDs, and considering the well-known logarithmic relationship between concentration and free energy, it is reasonable to hypothesize a linear correlation between the depth of the free-energy well associated with membrane translocation and the observed pEC_50_ values for the PCDDs series.

In Figure 6A, the free energy differences for membrane translocation, represented by the free energy minima from Figure 4, are plotted against the pEC_50_ values reported in Tuppurainen et al.^67^ Given that the free energy profiles appears to show an anomaly for the **m7** molecule, the free energy values obtained at the membrane center using the MM/GBSA method, for which such as abnormal behavior for **m7** is not observed, are also plotted against the pEC_50_ values in Figure 6B for comparison. Good linear correlations are found in both cases, with **m7** being the only outlier in Figure 6A. As will be discussed in the following section, the binding affinity behavior of PCDDs for the AhR lends further support to the hypothesis that differential membrane translocation represents the main factor of their relative toxic potencies.

**Figure 6:**
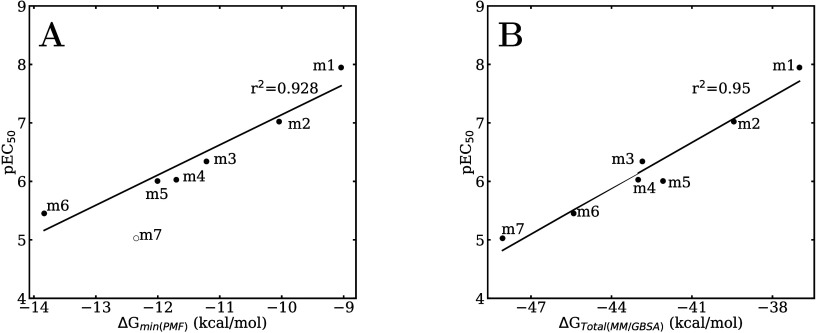
(A) pEC_50_ index vs the free energy minima of the membrane permeation. (B) pEC_50_ index vs MM/GBSA ligand/membrane binding free energy calculated at the membrane center.

The statistical QM/MM -EDA conducted at the membrane center for the various PCDDs provides deeper insight into the nature of the noncovalent interactions that govern their stability within the lipid environment. Additionally, these results offer a benchmark for evaluating the accuracy of classical force field predictions, particularly concerning overall interaction strength and the relative stability. Figure 5B and Table S2 report the mean ligand/membrane total interaction energies, Δ*E*_int_, and the different components, calculated over a set of representative equally spaced geometries sampled at the center of the membrane. Panels A and B of Figure 3 show the convergence analysis of the interaction energy and its components across the set of geometries and residues. The mean value of the total interaction energy in **m1** is −31.93 kcal/mol, progressively decreasing (i.e., becoming more stabilizing) with increasing chlorine substitution, reaching −37.80 kcal/mol for **m7**. These interaction energies correlate quite well with the classical MM/GBSA free energies, as shown in Figure 7A.

**Figure 7:**
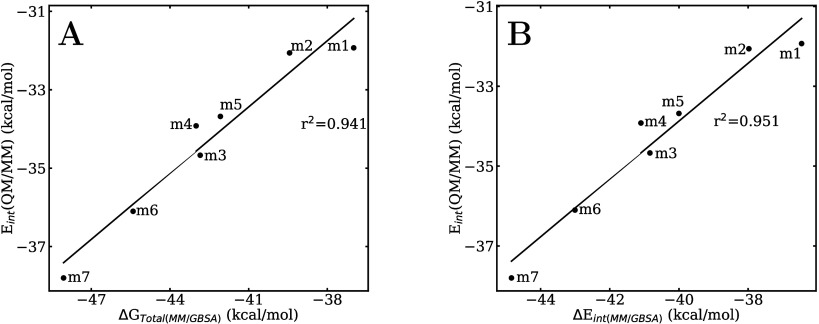
QM/MM total interaction energies vs (A) MM/GBSA ligand/protein binding free energy and (B) MM/GBSA total interaction energy.

However, the explicit inclusion of QM effects, which are absent from classical force fields, reveals important discrepancies in how specific noncovalent interactions are represented. Notably, while polarization dominates the interaction energy, the electrostatic component emerges as a non-negligible contributor to the stabilization of the pollutants in the membrane. In order to compare the electrostatic energy contribution against the non-electrostatic one (vdW), as it was done in the analysis of the classical energies, the vdW QM energy is computed as the sum of the Pauli and polarization terms, which is analogous to the vdW energy provided by the classical Lennard-Jones potential from the force field. When compared with the vdW QM energy, the electrostatic QM contribution shows a comparable or even slightly greater magnitude, particularly in the highly chlorinated species **m6** and **m7**. This stands in contrast to the classical MM/GBSA calculations, which largely underestimate the electrostatic contribution and, in the case of **m7**, even predict a slightly destabilizing effect.

Additionally, dispersion energy constitutes the most stabilizing contribution, accounting for approximately 69–70% of the total stabilization energy, followed by electrostatic (21–22%) and induction contributions (8–9%). These relative proportions remain quite invariant across the PCDDs series. As anticipated, all energetic components increase in absolute magnitude with progressive chlorination. Consequently, the enhanced energetic stability observed with increasing chlorine substitution is primarily due to a smaller relative increase in Pauli repulsion compared to the overall rise in attractive interactions, rather than to the amplification of any specific attractive component.

### AhR Binding

In the previous section, it was shown that the lower the free energy barrier for membrane permeation, the higher the toxicity of a given PCDD. Both classical and quantum results initially suggest that membrane translocation is the most critical step in determining toxicity within the studied series. These findings also indicate that translocation across the membrane is a thermodynamically unfavorable process, meaning that only a small fraction of PCDD molecules reach the intracellular space to interact with the AhR. The next question, therefore, concerns how differences in AhR binding affinity among the studied PCCDs influence their toxicological activity. This factor is anticipated to be critical, given that AhR activation requires ligand binding to the PCDD molecule.

As a first step, an MM/GBSA analysis was conducted to see if the binding energies correlate with the toxicity and to identify the key residues of AhR contributing to the binding free energy. As shown in Figure 8, phenylalanine at position 295 (PHE295) emerged as the most important interacting residue across all PCDDs, followed by another phenylalanine, PHE351. Eight additional residues exhibiting notable interactions with the pollutants are also presented in the figure. Notably, the MM/GBSA results align well with the AhR receptor cavity previously described in the literature for planar ligands,^58^ with all but PRO297 appearing among the top ten interacting residues. These ten residues have been selected for inclusion in the QM region of the QM/MM-EDA calculations as described above.

**Figure 8:**
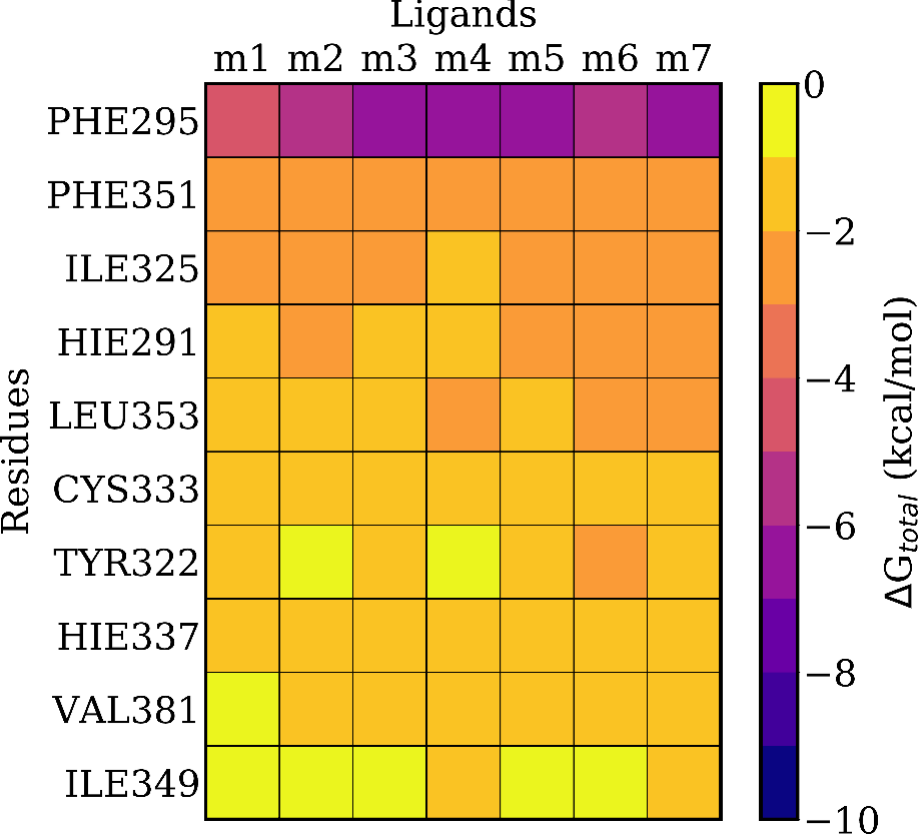
Pairwise residue decomposition of the ligand/protein binding free energy.

The free energy values, along with their decomposition into electrostatic, vdW, and solvation components using the MM/GBSA method, are presented in Figure 9A and Table S3. As observed in the membrane calculations, the electrostatic contribution appears to be significantly underestimated, contributing only marginally to the total pollutant/protein interaction energy. In contrast to the membrane results, however, the solvation component here is repulsive, resulting in total free energy values that are very similar to those obtained in the membrane. Specifically, the free energy in the membrane ranges from −37.0 kcal/mol in **m1** to −48.1 kcal/mol in **m7**, while in the protein, it ranges from −37.7 kcal/mol in **m1** to −49.3 kcal/mol in **m7**. In both environments, the free energy becomes progressively more favorable with an increasing number of chlorine substituents.

**Figure 9:**
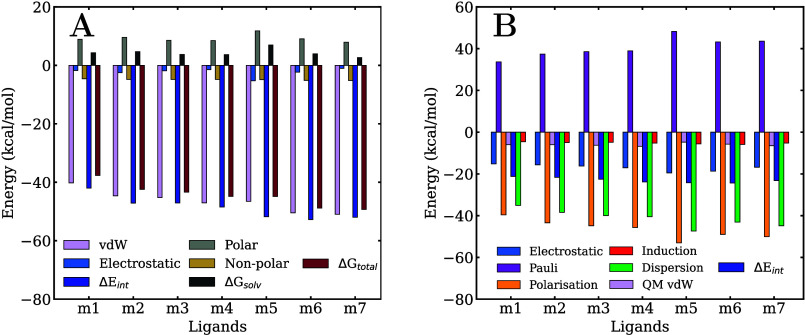
Decomposition of the ligand/protein (A) MM/GBSA binding free energy and (B) QM/MM-EDA total interaction energy.

These strongly stabilizing free energy values indicate that the binding of PCDDs to the AhR is highly thermodynamically favorable in all cases. This suggests that even the small fraction of molecules that permeate the cell membrane will readily bind to the receptor—or to other biological targets with higher affinity—potentially masking the specific contribution of AhR binding to the relative toxicity of different PCDDs. In addition, the finding that increased chlorination enhances binding affinity to the AhR, as indicated by more negative binding free energies, leads to an inverse correlation between binding free energies and pEC_50_ values. This initially seems contradictory, as receptor activation is generally assumed to require ligand binding. However, this apparent paradox may be explained by recognizing that while receptor binding is a prerequisite for activation, it is not the sole determinant in the action mechanism. Highly chlorinated ligands, despite their strong affinity, may impair the receptor’s conformational flexibility or interfere with subsequent steps in the activation cascade, such as nuclear translocation, dimerization with ARNT, or transcriptional complex assembly. These mechanistic constraints suggest that a high degree of chlorination may decouple binding from functional activation, thereby weakening the predictive power of affinity alone in toxicodynamic assessments.^68^

To further explore the nature of the binding between PCDDs and the AhR, QM/MM-EDA calculations were performed on a series of equally spaced geometries sampled from the production phase of the CMD simulations. The mean values of the ligand/protein total interaction energy, Δ*E*_int_, and its individual components are summarized in Figure 9B and Table S4. On the other hand, Figure 3C presents the convergence analysis of the interaction energy and its components across the sampled geometries. The QM region employed for the calculations encompassed the ten amino acids that interact most strongly with the PCDD ligands, based on the MM/GBSA analysis presented in Figure 8.

According to the QM/MM-EDA calculations, the total interaction energy generally increases with the number of chlorine atoms, similar to the trend observed in the membrane, though the difference between ligands is about half of that seen in the membrane. The interaction energy ranges from −21.2 kcal/mol in **m1** to −24.4 kcal/mol in **m6**. However, **m7** deviates from this trend, exhibiting an interaction energy (in absolute value) lower than that of **m4**, **m5**, and **m6**. This deviation may reflect an insufficiently sized QM region for this molecule, suggesting the need for a larger number of amino acids to more accurately represent the interaction at the quantum level. In fact, **m7** is the pollutant that shows the strongest classical interactions with the ten amino acids selected for the QM region, as indicated by the color scale in Figure 8. This suggests that some important amino acids may be missing from the QM region, leading to discrepancies in the predicted interaction energy compared to the correct value. Consequently, the expected energy order may be altered, as the differences between pollutants are very small in this case.

The interaction energy components presented in Figure 9B and Table S4 account for the differences observed in the total energies with respect to the membrane. While the electrostatic energies are similar to those calculated in the membrane, especially for the least chlorinated compounds, the polarization energies in AhR are much less negative, and this reduction is not compensated by a corresponding decrease in Pauli energy. Dispersion energy remains the most stabilizing component, but its values in AhR are notably lower than those observed in the membrane because the center of the membrane is much more nonpolar than the binding pocket of the AhR protein. The induction energy continues to be the least significant stabilizing term, and, like the electrostatic energy, the differences with the membrane are relatively small.

Since the toxicity correlates well with the energy needed to cross the membrane but does not correlate with the binding energy to the AhR protein, it seems that membrane permeation is the relevant step, although binding to the protein is obviously necessary. However, this binding is very favorable for all ligands investigated here.

## Concluding Remarks and Future Perspectives

This work provides compelling evidence that membrane translocation constitutes the central determinant of the relative toxicity of PCDDs. The progressive decrease in free energy with increasing chlorination, together with the linear correlation between permeation energies and experimental pEC_50_ values, supports the notion that intracellular accumulation critically regulates toxic potency. Regarding AhR binding, our results confirm that ligand–receptor association is thermodynamically highly favorable across all congeners and becomes progressively stronger with chlorination. Nevertheless, the observed inverse relationship between binding free energy (and interaction energy) and pEC_50_ values suggests that toxicity cannot be explained solely by receptor recognition. In this sense, highly chlorinated congeners may exhibit strong binding but limited activation, reducing the predictive power of AhR binding affinity alone.

Although a reasonably good correspondence is observed between the quantum and classical total interaction energies, pronounced discrepancies are observed in the contribution of electrostatic interactions to the stabilization of PCDDs. Specifically, within both the membrane environment and the AhR binding pocket, the classical model markedly underestimates the electrostatic component. This underestimation is especially problematic in membrane-permeation studies involving zwitterionic phospholipids, such as the one presented here, because it introduces significant errors that prevent classical methods from providing reliable insight into the intermolecular interactions governing membrane transport. These discrepancies underscore the need for hybrid approaches that incorporate quantum effects when characterizing noncovalent interactions relevant to the toxicity of these compounds.

Despite the computational complexity of the biological systems analyzed here, future work should aim to incorporate entropic contributions and dynamical factors into affinity assessments at the hybrid QM/MM level, as well as to extend the analysis to additional nuclear receptors, thereby providing a more comprehensive framework for predicting the toxicity of persistent halogenated pollutants.

## Supplementary Material



## Data Availability

Data for this article, including input files for molecular dynamics simulations, QM/MM calculations, and energy decomposition analyses for the pollutant/membrane and pollutant/protein systems are available at the Zenodo repository at DOI: 10.5281/zenodo.17339579 (https://zenodo.org/records/17357729).

## Notes

(Figures S1 and S2) Convergence analysis of the umbrella sampling simulations; (Tables S1) ligand/membrane binding free energies and calculated contributions; (Table S2) ligand/membrane total interaction energies and contributions; (Table S3) ligand/protein binding free energies and calculated contributions; (Table S4) ligand/protein total interaction energies and contributions (PDF)
