# Supplementary material for: Unravelling the Role Played by Non-covalent Interactions in the Action Mechanism of PCDDs within Cells
Source: J Chem Inf Model. 2026 Jan 29;66(3):1801–12. doi: 10.1021/acs.jcim.5c02555 (PMC12892313; doi:10.1021/acs.jcim.5c02555)
Supplement: Supplementary file 1 [file ci5c02555_si_001.pdf]

Supporting Information
Unravelling the Role Played by Non-Covalent Interactions in the Action Mechanism of PCDDs within Cells.

Lorena Ruano,†,‡ Álvaro Pérez-Barcia,‡ Vito F. Palmisano,†,¶ Juan J. Nogueira,*,†,§ Marcos Mandado,*,‡ and Nicolás Ramos-Berdullas*,‡

†*Department of Chemistry, Universidad Autónoma de Madrid, 28049, Madrid, Spain.*

‡*Department of Physical Chemistry, University of Vigo, Lagoas-Marcosende s\n, ES-36310-Vigo, Galicia, Spain*

¶*Theoretical Chemistry Group, Zernike Institute for Advanced Materials, University of Groningen, Groningen, The Netherlands.*

§*Institute for Advanced Research in Chemistry (IAdChem), Universidad Autónoma de Madrid, 28049 Madrid, Spain*

E-mail: juan.nogueira@uam.es; mandado@uvigo.es; nicolas.ramos@uvigo.es

This Supporting Information file contains the test done for the selection of the umbrella sampling constant (Figure S1); the convergence analysis of the PMF (Figure S2); the ligand/membrame and ligand/protein binding free energies and their decomposition in Tables S1 and S3, respectively; and the total interaction ligand/membrane and ligand/protein energies and their decomposition in Tables S2 and S4, respectively.

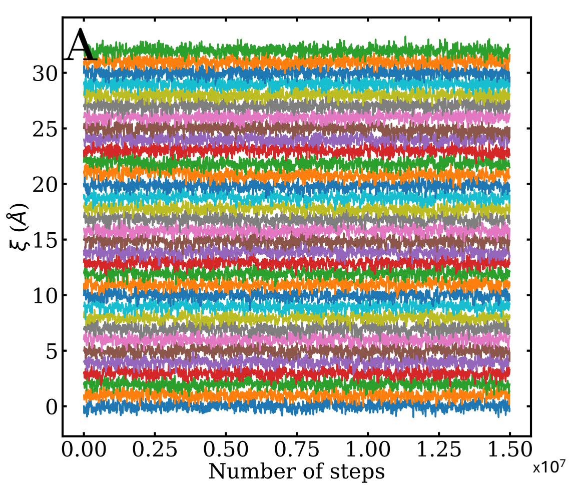

Figure S1: Tests to check whether the chosen force constant and number of windows of the umbrella sampling simulation are adequate. Results for **m1** ligand. A) Ligand position along the simulation for all windows. B) Reaction coordinate probability distributions for windows.

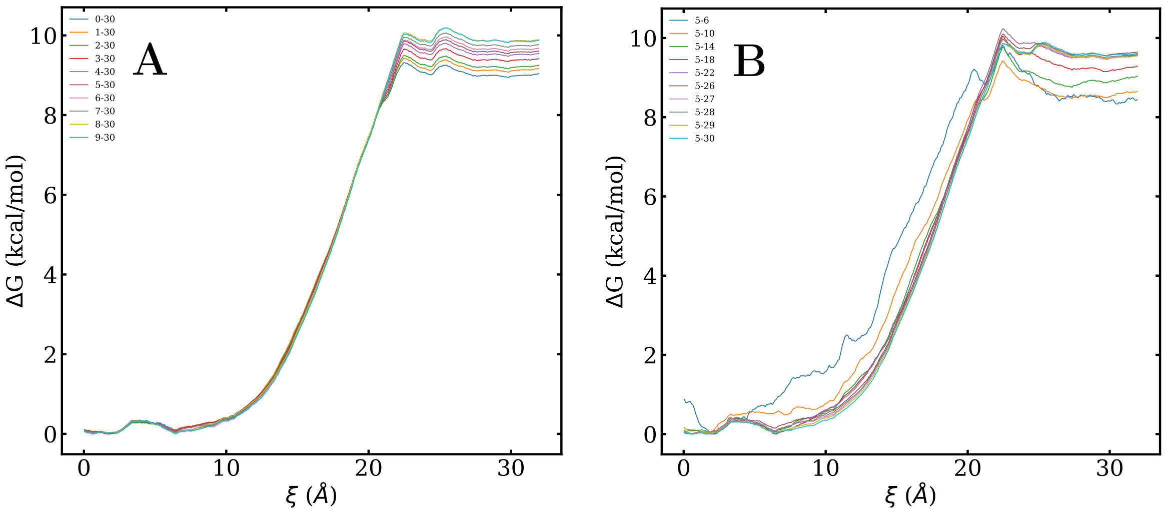

Figure S2: Convergence analysis of the umbrella sampling simulations of **m1** ligand. A) Potential of mean force computed by removing different time intervals from the beginning of each window. B) Potential of mean force computed for different computational times from the initial time of 5 ns.

Table S1: Ligand/membrane binding free energy ΔG_{total} decomposition into vdW, electrostatic, and solvent polar and non-polar contributions by means of the MM/GBSA method. The ligand/receptor interaction energy ΔE_{int} is the summation of the vdW and electrostatic contributions, while the solvation free energy ΔG_{solv} accounts for the polar and non-polar contributions. All energies are in kcal/mol. Standard deviations are included.

	**vdW**	**Electrostatic**	**Polar**	**Non-polar**	**ΔE_{int}**	**ΔG_{solv}**	**ΔG_{total}**
**m1**	-36.19±2.31	-0.19±0.41	4.26±0.53	-4.86±0.19	-36.39±2.37	-0.60±0.50	-36.99±2.37
**m2**	-37.82±2.43	-0.15±0.43	3.55±0.48	-5.01±0.20	-37.98±2.47	-1.46±0.50	-39.44±2.63
**m3**	-40.67±2.41	-0.17±0.38	3.16±0.44	-5.17±0.19	-40.84±2.47	-2.01±0.46	-42.85±2.64
**m4**	-40.78±2.47	-0.32±0.50	3.27±0.51	-5.18±0.18	-41.10±2.57	-1.90±0.53	-43.00±2.66
**m5**	-39.81±2.48	-0.19±0.49	3.03±0.56	-5.11±0.20	-40.00±2.50	-2.08±0.58	-42.08±2.69
**m6**	-42.72±2.45	-0.29±0.45	2.88±0.55	-5.29±0.20	-43.01±2.54	-2.40±0.60	-45.41±2.74
**m7**	-44.89±2.81	0.04±0.27	2.20±0.46	-5.42±0.21	-44.84±2.81	-3.22±0.53	-48.06±3.14

Table S2: Ligand/membrane total interaction energy decomposition into electrostatic, Pauli, and polarisation (dispersion + induction) by means of the QM/MM-EDA approach. All energies in kcal/mol. Standard deviations are included.

	**Electrostatic**	**Pauli**	**Polarisation**	**ΔE_{int}**	**Dispersion**	**Induction**	**QM vdW**
**m1**	-16.67±3.10	46.16±8.85	-61.44±7.37	-31.93±3.66	-54.61±6.22	-6.84±1.32	-15.28±4.16
**m2**	-18.64±4.17	51.67±11.51	-65.11±8.54	-32.06±3.03	-57.96±7.20	-7.15±1.47	-13.44±4.50
**m3**	-19.38±4.31	53.31±11.31	-68.62±8.32	-34.67±2.60	-61.21±7.01	-7.41±1.46	-15.31±3.98
**m4**	-17.54±3.10	48.20±8.96	-64.60±6.98	-33.92±3.04	-57.75±6.01	-6.85±1.10	-16.40±4.06
**m5**	-18.62±3.46	50.82±9.77	-65.91±8.55	-33.68±3.54	-58.93±7.26	-6.97±1.41	-15.08±3.42
**m6**	-20.20±4.57	54.88±12.50	-70.80±9.17	-36.10±2.68	-63.49±7.83	-7.31±1.46	-15.92±4.33
**m7**	-21.57±4.44	59.56±12.07	-73.52±15.91	-37.80±2.90	-67.89±7.71	-7.93±1.45	-15.77±5.09

Table S3: Ligand/protein binding free energy ΔG_{total} decomposition into vdW, electrostatic, and solvent polar and non-polar contributions by means of the MM/GBSA method. % residues is the contribution of the top 10 interacting residues to the ΔG_{total}. The ligand/receptor interaction energy ΔE_{int} is the summation of the vdW and electrostatic contributions, while the solvation free energy ΔG_{solv} accounts for the polar and non-polar contributions. All energies are in kcal/mol. Standard deviations are included.

	**vdW**	**Electrostatic**	**Polar**	**Non-polar**	**ΔE_{int}**	**ΔG_{solv}**	**ΔG_{total}**	**% residues**
**m1**	-40.21±1.86	-1.78±0.92	8.89±0.68	-4.59±0.11	-41.99±1.96	4.31±0.66	-37.69±1.89	46
**m2**	-44.64±1.85	-2.50±0.90	9.56±0.73	-4.84±0.11	-47.14±2.00	4.72±0.73	-42.42±1.93	48
**m3**	-45.25±2.56	-1.84±0.92	8.54±0.80	-4.81±0.14	-47.09±2.77	3.73±0.78	-43.36±2.64	49
**m4**	-47.05±2.44	-1.45±1.06	8.49±0.82	-4.82±0.17	-48.50±2.71	3.67±0.80	-44.83±2.63	46
**m5**	-46.51±2.29	-5.28±1.31	11.81±1.24	-4.85±0.16	-51.79±2.79	6.95±1.20	-44.83±2.39	48
**m6**	-50.44±1.97	-2.31±0.74	9.07±0.60	-5.14±0.10	-52.76±2.17	3.93±0.62	-48.83±2.09	45
**m7**	-50.96±2.56	-1.04±0.73	7.89±0.70	-5.22±0.19	-52.00±2.48	2.67±0.81	-49.33±2.75	47

Table S4: Ligand/protein total interaction energy decomposition into electrostatic, Pauli, and polarisation (dispersion + induction) by means of the QM/MM-EDA approach. All energies in kcal/mol. Standard deviations are included.

	**Electrostatic**	**Pauli**	**Polarisation**	$\Delta \mathbf{E}_{int}$	**Dispersion**	**Induction**	**QM vdW**
**m1**	-15.23±2.48	33.67±5.89	-39.64±3.93	-21.19±2.04	-35.05±3.46	-4.60±0.71	-5.97±2.77
**m2**	-15.63±3.41	37.36±7.86	-43.41±5.37	-21.66±2.42	-38.40±4.64	-5.01±0.99	-6.05±3.25
**m3**	-16.25±3.37	38.53±8.53	-44.85±6.02	-22.55±2.27	-40.00±5.35	-4.85±0.93	-6.32±3.31
**m4**	-17.06±3.71	38.92±9.76	-45.71±6.94	-23.83±2.98	-40.48±5.97	-5.23±1.15	-6.79±3.72
**m5**	-19.51±3.31	48.21±8.52	-52.99±5.11	-24.27±1.83	-47.39±4.57	-5.61±0.75	-4.78±3.90
**m6**	-18.67±3.51	43.23±8.59	-48.96±6.13	-24.38±2.82	-43.07±5.42	-5.89±0.91	-5.73±3.57
**m7**	-16.79±3.46	43.57±8.42	-50.03±5.45	-23.22±1.78	-44.83±4.75	-5.20±0.83	-6.45±3.45
